# Enriching sociocultural perspectives on the effects of idealized body norms: Integrating shame, positive body image, and self-compassion

**DOI:** 10.3389/fpsyg.2022.983534

**Published:** 2022-11-25

**Authors:** Jennifer S. Mills, Claire Minister, Lindsay Samson

**Affiliations:** Department of Psychology, York University, Toronto, ON, Canada

**Keywords:** sociocultural model, body image, body shame, self-compassion, social comparison

## Abstract

Body image is an integral aspect of the psychology of the self. Idealized body images are ubiquitous in both traditional media forms (e.g., magazines, television) and social media (e.g., Facebook, Instagram). The classic sociocultural model of body image (i.e., the Tripartite Influence Model) emphasizes pathways between idealized body norms, appearance comparisons, internalization of body ideals, and body dissatisfaction and its outcomes. We summarize the model and identify some issues to be addressed in future work, particularly in light of the immense popularity of social media. We review three topics that are not included in the sociocultural model but that provide a more complete picture of the influence of societal body norms on body image: (1) body shame, (2) positive body image, and (3) self-compassion. Research on the nature, assessment, and relevance of these constructs is reviewed in detail. In terms of clinical applications of these areas of research for individuals at risk of body dissatisfaction, we suggest assessing for and targeting body shame, cultivating facets of positive body image, and teaching strategies for developing self-compassion.

## Introduction

Body image is defined as a multifaceted psychological construct relating to embodiment (i.e., the complex experience of living in the body) (Piran, 2016), It includes perceptual (i.e., judgments about the size or shape of one's body), attitudinal (e.g., internalized pressure to be thin), cognitive (i.e., thoughts and beliefs about one's body), evaluative (i.e., positive and/or negative feelings about one's body), and behavioral components (e.g., food restriction) (Cash, 2004).

Body image develops in the context of an awareness of societal norms regarding body types that are considered to be attractive and desirable. Although body norms are sometimes conceptualized in terms of the perceived body preferences of other people (Bair et al., 2014) or as the perceived average body size of a population (Mills et al., 2012), most of the literature on body image and the influence of societal body norms focuses on idealized body types. These norms inform the body that people want to have and feel they should have in order to feel happy, confident, and attractive. The “thin-ideal” body is consistently glorified for women and represents a slim female body with a small waist and little body fat (Low et al., 2003). The idealized body type for men is tall, muscular, and lean (Ridgeway and Tylka, 2005). Both female and male bodies that are seen as having “excess” fat on them are widely denigrated in Westernized cultures. In recent years, variations on the thin-ideal for women have emerged. These include a toned and fit body (the “fit-ideal”) as well as a curvier body known as the “slim-thick ideal,” characterized by a very small waist but with wide hips and large buttocks (McComb and Mills, 2022). Body ideals for both men and women are consistently represented by body types that are rare and difficult to attain.

It is well-documented that idealized body norms can lead to body image disturbance and unhealthy behaviors, including dieting and clinically disordered eating. In the next section of the article, we provide a brief overview of the body of research that has led to those conclusions. We identify some notable gaps in the current state of the literature on idealized body norms. In the remainder of the article, we review in detail some different perspectives on body image that we consider to be important for enriching sociocultural perspectives on body image. Specifically, we review the relevant literature on body shame, positive body image, and self-compassion, and discuss some of the applications of those topics for both body image research and for clinical work with individuals experiencing body image distress.

## The sociocultural model of body image

The Tripartite Influence Model (Thompson et al., 1999) is a highly influential theory of body image and is often referred to simply as “the sociocultural model” of body image. It was one of the first theoretical approaches to the study of body image to emphasize the role of idealized body norms in the pathway to body image distress and impairment. The theory proposes that body ideals are communicated to people throughout their life by peers, family, and the media (originally in the forms of traditional media and now also in social media). Through each of these three sources, people become aware of idealized body norms. As a result of both internalizing these ideals and by comparing oneself to them, body dissatisfaction occurs. Comparing oneself to highly attractive and internally idealized bodies results in a negative evaluation of one's own body (e.g., “feeling fat”). Body dissatisfaction can manifest as negative perceptions, cognitions, or feelings about one's body. In other words, the interplay between societal body ideals, an intrinsic drive to evaluate ourselves relative to other people (Festinger, 1954), and personality leads to negative body image. Body dissatisfaction, in turn, can lead to dieting, bulimic symptoms, and other forms of psychological distress, such as depression.

**Figure 1:**
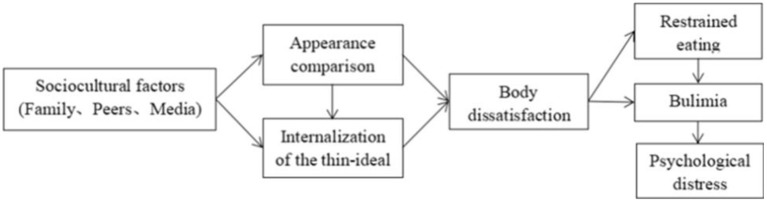
The Tripartite Influence Model of body image based on Thompson et al. (1999).

An illustration of the sociocultural model of body image appears in Figure 1.

This classic sociocultural model is well-supported by decades of research. Overall, it continues to offer a useful framework for much of the research done in the area of body image today. Societal body ideals are ubiquitous and easily identified among women (McComb and Mills, 2022), including the newer variations on the thin-ideal, such as the fit-ideal and the slim-thick ideal noted above. There is relatively less research in men's body image ideals and how they may be evolving, but there is no evidence to suggest that they have changed from having an emphasis on height, leanness, and musculature (McCreary, 2012). There is substantial evidence that societal norms around beautiful bodies elicit appearance comparisons (Mills et al., 2017). Given the extreme nature and rarity of idealized body types in the real world, a perceived discrepancy between an idealized body and what regular people actually look like is commonplace (Mills et al., 2017). As proposed by the model, a key determinant to whether body dissatisfaction results from exposure to idealized body norms is the extent to which an individual internalizes the personal importance of an idealized body type. For example, qualitative research reveals that internalization of the message that being thin will make you happier, more desirable, and more successful in life is crucial in determining whether women feel affected by images of the thin-ideal body type (Ahern et al., 2011). Interestingly, a study (Ashikali and Dittmar, 2010) comparing congenitally blind and sighted women found that the blind women reported less body dissatisfaction and dieted less than did sighted women, suggesting that actually seeing idealized body images is key to feeling as if one does not measure up. In the same study, body image attitudes, particularly thin-ideal internalization, accounted for differences in body dissatisfaction and dieting among blind women, consistent with the sociocultural model. Other overlapping personality traits indirectly support the pathways in the sociocultural model. For example, having high levels of appearance-related perfectionism (e.g., “I want my body to look perfect”) along with tendency toward appearance comparisons predicts body dissatisfaction after looking at thin-ideal images on social media (McComb and Mills, 2021). Likewise, in experimental studies, exposure to images of idealized bodies on social media has been shown to lead to increased state-based negative mood and body dissatisfaction as well as increased desire to undergo cosmetic enhancements (e.g., Brown and Tiggemann, 2016; Prichard et al., 2020; Walker et al., 2021). Body dissatisfaction as a result of appearance comparisons is a robust predictor of body surveillance and behaviors aimed at controlling one's weight, such as dieting and purging, and psychological distress (Stice and Shaw, 2002; Keery et al., 2004; Polivy et al., 2020). A systematic review concluded that, consistent with the sociocultural model of body image, engagement with image-related media content is positively related to body image concerns and disordered eating in both women and men, and that this relationship is mediated by internalization and appearance comparison (Holland and Tiggemann, 2016). In sum, there is a substantial body of literature to suggest that body norms idealized by society shape people's body image and associated behaviors.

### Issues to be addressed in the sociocultural model

A surge in published research on social media and body image in the past decade is further evidence of the enduring importance of the sociocultural model and the significant impact of idealized body norms on body image. Nevertheless, there are several gaps in research on the sociocultural model of body image that should be addressed in future research. Some research has found that negative self-evaluation does not always result from appearance comparisons to idealized body images, unlike what is predicted by the sociocultural model. Some women experience inspiration or motivational drive to emulate the ideal (Mills et al., 2002; Tiggemann and Polivy, 2010). The need to better understand people's motives for viewing idealized body images was raised almost 10 years ago by Perloff (2014) amidst the rising popularity of social media. However, little progress has been made in understanding why people choose to expose themselves to images that make them feel bad about their body. It would be worthwhile for future research to explore people's motives behind self-selected exposure to idealized body norms since that it how so much of today's media content is consumed.

Social media use allows people some degree of control over whether they choose to view idealized body images and how they engage with those images. A recent paper by Vandenbosch et al. (2022) describes in detail some of the unique features of social media and how they impact body image. Nowadays, social media and appearance-based applications produce an almost endless stream of content. The frequency of exposure to idealized body images is an aspect of social media that makes it particularly influential (Hogue and Mills, 2019). Exposure to idealized body images can now potentially occur throughout a person's day, depending on how much social media they use and how they engage with it. A meta-analytic review determined that there was a stronger relationship between appearance-based social media and internalization of the thin ideal than overall time spent on social media (Mingoia et al., 2017). Updated and more specific recommendations around how to mitigate the risk of body dissatisfaction brought about by social media would be helpful. To this end, it may be useful to look to research on the topic of social media and depressive symptoms. A recent meta-analysis (Cunningham et al., 2021) examined how level of social media use impacted adverse mental health symptoms in youth. Time spent on social media and the intensity of social media use (e.g., number of accounts followed, emotional connection to social media) both had statistically significant but weak correlations with depressive symptoms. On the other hand, it was problematic use (e.g., not feeling able to control one's use of social media, functional impairment caused by social media) that was the strongest correlate of depressive symptoms, and that correlation was significantly greater than the relation between depressive symptoms and the other two measures of social media use. Applied to body image research, it could be that time spent on social media is not as strong an influence on body image as more active and emotional engagement with appearance-based social media, as operationalized by commenting on other people's posts and editing one's own photos, and compulsive appearance comparisons and/or body checking behaviors. Future research should aim to identify more precisely what constitutes problematic social media use when it comes to body image.

The sociocultural model of body image focuses on body dissatisfaction and its outcomes related to health. An emerging area also under study is the outcome of unnecessary or cosmetic surgery and its relation to social media use and body image. One study (Walker et al., 2021) found that participants who saw cosmetically enhanced females (vs. neutral images) and who were heavy users of social media were significantly more likely to desire cosmetic surgery. Interestingly, the results showed that the relationship between social media use and desire for cosmetic surgery was not mediated by body dissatisfaction. Social media use was a stronger predictor of a participants' desire for cosmetic surgery than body dissatisfaction. More research is needed to determine whether desire for cosmetic surgery is, in fact, a result of body dissatisfaction resulting from appearance comparisons to idealized body norms. If so, elective surgery could be added to the list of potentially unhealthy (or at least risky) outcomes of body dissatisfaction in the sociocultural model. However, the research described above raises doubts about whether a desire for cosmetic surgery is significantly related to idealized body norms. It could be that exposure to persuasive advertisements on social media (e.g., before and after photos) that elicits a desire for surgery, but not necessarily through appearance comparisons and resulting body dissatisfaction.

We now turn to a thorough review of three topics that we argue can enrich the sociocultural model. We consider body shame, positive body image, and self-compassion. These subjects are all absent from the classic sociocultural model of body image but their emerging fields of research suggest that they are necessary to develop a more complete understanding and offer important perspectives that balance the model.

## Body shame

As reviewed above, the sociocultural model of body image elucidates social and individual predictors of body dissatisfaction and associated behaviors. Another consequence of feeling as if one does not measure up to societal ideals around attractiveness is the reaction of shame. Shame can be defined as a complex emotional response consisting of feelings such as anger, sadness, disgust, and feeling as if one is fundamentally and substantially flawed or defective (Gilbert, 2011). In this section, we discuss shame, what is known about its relation to body image, how it may be applied to the sociocultural model, and the similarities and differences between body shame and body dissatisfaction.

Shame can be broken down into two components: external and internal shame. External shame is focused on how one is perceived in the mind of others. For example, one might believe that others perceive them as disgusting, inadequate, or worthy of ridicule, which would be accompanied by the social threats of rejection, marginalization, or persecution (Gilbert, 2011). Conversely, internal shame occurs within oneself, consisting of negative feelings, judgments, and criticism directed at the self (Gilbert, 2011). External shame is understood to have an evolutionary basis. That is, people desire to be cared for, be well-regarded socially, and be seen as valuable, because throughout evolution, these things were tied to survival, as one's social group might not protect them or may even become hostile toward them if they do not like or care for them (Gilbert, 2011). Thus, shame is evolutionarily adaptive as it serves as a reminder that one must conform in order to fit in with the group and ensure their survival.

Body shame specifically can be defined as feelings of shame (i.e., disgust, anger, etc.) directed toward one's body, either externally (i.e., focused on other people's perception of one's body) or internally (i.e., internal criticism of one's body). Both body shame and general shame have been linked to eating disorders (e.g., Goss and Allan, 2009; Troop and Redshaw, 2012), body image disturbance (e.g., Duarte et al., 2015), and eating behaviors (e.g., Swan and Andrews, 2003). Indeed, a recent meta-analysis (Nechita et al., 2021) concluded that general shame, and body and eating shame in particular, are significantly positively associated with eating disorder symptoms. Most research studies in this area are cross-sectional, and thus further study of the link between different forms of shame (e.g., external, internal, body-focused, eating focused) and their impact on different types of eating disorder symptoms (e.g., dietary restriction, binge eating) using robust methods (e.g., longitudinal methods, ecological momentary assessment) is warranted (Nechita et al., 2021). In service of furthering research in this area, a number of shame measures are currently available in the literature. For instance, general shame can be measured by the Experience of Shame Scale (Andrews et al., 2002), and a modified version (Swan and Andrews, 2003) can be used to assess eating shame specifically. External shame can be measured using the other as Shamer Scale (Goss et al., 1994), and internal/external body shame can be measured using the Body Image Shame Scale (Duarte et al., 2015).

Regarding the potential role of shame in the sociocultural model, given the previous discussion of the importance of internalization of body ideals and appearance comparison to body image and eating behavior, it is important to note that these processes are also implicated in creating body shame (Goss and Allan, 2009), just as they are implicated in creating body dissatisfaction and associated outcomes. To better understand body shame in particular, two foundational theories to this area of work should be considered: objectified body consciousness (McKinley and Hyde, 1996) and self-objectification theory (Fredrickson and Roberts, 1997). These are related feminist theories which essentially state that from a young age, girls and women are viewed as sexual objects for the pleasure of those around them, particularly men. Thus, women are socialized into learning to view themselves from an outside perspective (i.e., they learn to engage in self-objectification) and subsequently learn to monitor and evaluate their attractiveness (i.e., engage in body surveillance) to determine whether they are meeting the societal standards for female beauty. According to these theories, when women self-objectify and find themselves lacking in attractiveness compared to others, it brings about feelings of shame. Self-objectification and body surveillance share features with the processes of appearance comparison and thin ideal internalization (i.e., the mechanisms theorized to maintain the sociocultural model of body image leading to disordered eating; see Figure 1). Specifically, these processes involve viewing oneself from an outside perspective, or the perspective of others (self-objectification, body surveillance), to determine whether one's attractiveness measures up to societal ideals (i.e., body surveillance) and/or to the attractiveness of those around them (i.e., appearance comparison), all of which occurs because of the objectification of women in society and the resulting internalization of idealized female bodies. Together these factors create an environment where shame can occur, which may ultimately result in disordered eating.

Although research in this area remains nascent, there is evidence that shame may fit into the sociocultural model as a mechanism in several different relations. First, there is evidence that body shame may either precede or follow body dissatisfaction. For instance, in a sample of adolescent girls and boys, Knauss et al. (2008) found that body surveillance (similar to appearance comparison) predicted body shame, which subsequently predicted body dissatisfaction, whereas Mustapic et al. (2015) found that body shame partially mediated the relation between body dissatisfaction and disordered eating behaviors in a sample of adolescent girls. Second, there is evidence that shame itself is related to eating behaviors independent of body dissatisfaction (i.e., the key mechanism responsible for disordered eating in the sociocultural model). For example, in a community sample of young women, Andrews (1997) found that bodily shame was uniquely associated with bulimia symptoms independent of body dissatisfaction. Similarly, Fredrickson et al. (1998) found that body surveillance predicted body shame, which subsequently predicted restrained eating in women, but not men. In a female undergraduate sample, Noll and Fredrickson (1998) found that shame mediated the relation between self-objectification and disordered eating, and Melioli et al. (2015) found that the relation between time spent on the internet and bulimia symptoms was mediated by body shame, but not body dissatisfaction or self-surveillance. Lastly, other researchers have conceptualized shame as a mechanism among other relations in the sociocultural model. For instance, in samples of female undergraduates, Markham et al. (2005) found that appearance comparison mediated the relation between thin ideal internalization and body-image shame, and Calogero (2009) found that body shame mediated the relation between self-objectification and disordered eating. Taken together, research suggests that shame may be an important mechanism to consider incorporating into the sociocultural model in order to better account for the emotional impact of these processes than body dissatisfaction alone.

Given that research has highlighted the importance of both body shame and body dissatisfaction as risk factors for disordered eating, it is important to understand the role of each. However, body shame and body dissatisfaction have not been explicitly delineated in the research literature, even though both consist of negative evaluations of one's body that can lead to disordered eating. Cash describes body image as consisting of two dimensions. The first is evaluation/affect, which consists of cognitive appraisal of the body, satisfaction with the body, and emotional responses related to that evaluation (Cash, 2004). The second component is investment, or how central shape and weight concerns are to a person's self-evaluation, including how much attention is paid to managing one's physical appearance. By this view, body dissatisfaction would likely represent the evaluative component of body image. Indeed, a recent systematic review of body image instruments places body satisfaction/dissatisfaction in the evaluation/affect component of body image (Kling et al., 2019). Regarding body shame, Gilbert (2011) describes shame as a complex secondary self-conscious emotion consisting of primary emotional components (e.g., disgust, anger), behavioral components (e.g., urges to run away and hide, avert eye contact), a negative or critical internal sense that the self is flawed, beliefs that others think of one negatively or as flawed or bad, and uncomfortable physiological sensations that are not yet well-understood. In addition to being intrapersonal, shame is also an interpersonal process, wherein people can overtly or covertly shame others (e.g., weight stigma; Gilbert, 2014).

There are many similarities between body dissatisfaction and body shame. For example, both body dissatisfaction and body shame can be variably conceptualized as a psychological trait (i.e., how someone usually feels) or a state (i.e., how someone feels in the moment). Both are informed by sociocultural factors (e.g., media, peers) and involve the processes of social comparison and internalization of some ideal for how the body should look (e.g., Gilbert et al., 1995; Myers and Crowther, 2009; Gilbert, 2014; Goss and Gilbert, 2014). All of these processes can ultimately result in finding oneself lacking in attractiveness compared to others, bringing about negative emotional responses like body dissatisfaction and/or shame.

In terms of differences between the two constructs, Gilbert (2014) emphasizes that although believing ones' body is unattractive is related to both shame and body dissatisfaction, an important distinction is that body shame is often linked to body function in addition to appearance (Gilbert, 2014), whereas body dissatisfaction seems to be uniquely tied to appearance. Another important distinction is that body shame involves consideration of how one is evaluated in the minds of others as a central process, whereas body dissatisfaction appears to be conceptualized more as self-focused discontentment. There are also research findings that speak to body shame and body dissatisfaction having unique effects. For example, Melioli et al. (2015) found that the relation between time spent on the internet and bulimia symptoms was mediated by body shame but not body dissatisfaction. Similarly, in a sample of young women, Andrews (1997) found that body dissatisfaction was more common than body shame, and that the relation between shame and bulimia symptoms was not explained by body dissatisfaction. Andrews (1997) concluded that one can experience body dissatisfaction without necessarily experiencing a shame component. Consistent with Andrews' conclusion, body dissatisfaction is very prevalent, particularly among young women, and has been referred to as “normative discontent” (e.g., Rodin et al., 1984; Cash and Henry, 1995). Although estimates differ, a 2014 review found that 11–72% of adult women report body dissatisfaction (Fiske et al., 2014), and a 2014 study found that 13.4–31.8% of U.S. women report body dissatisfaction using established clinical cutoffs on several body image measures (Fallon et al., 2014). Similar statistics do not seem to be available for the prevalence of body shame in the general adult population. Thus, whereas body dissatisfaction is very common, body shame, which is a more complex emotional, behavioral, and physiological phenomenon, could be rarer, more dangerous, and even more strongly related to psychological distress. More research is needed to disentangle the constructs of body shame and body dissatisfaction, and particularly to better understand the complex construct of shame and how it relates to disordered eating and eating disorders, in order to target shame more precisely using psychological interventions.

## Positive body image

Conceptually different from a negative evaluation of one's physical appearance (e.g., body dissatisfaction), positive body image is a multifaceted construct that has been defined as a respect and love for one's body regardless of whether aspects of the body are incongruous with societal ideals (Wood-Barcalow et al., 2010; Tylka, 2018). As has been discussed in this article, the highly influential sociocultural model of body image focuses on negative body image, with positive body image often operationalized as the absence of, or opposite of, body image disturbance (Thompson et al., 1999; Cash, 2002). There was no controversy around the concept of positive body image; there was simply little attention paid to it within the theoretical frameworks underlying body dissatisfaction and disordered eating. However, in the last two decades there has been a pivotal shift in the academic community toward exploring the positive and protective aspects of body image, resulting in a wealth of literature and an updated, comprehensive conceptualization of positive body image (Tylka and Wood-Barcalow, 2015b). Qualitative and mixed-methods studies have helped to formalize and expand the dimensionality of this construct (e.g., Frisén and Holmqvist, 2010; Wood-Barcalow et al., 2010), while quantitative studies have evidenced positive body image as a distinct construct from negative body image (Halliwell, 2015). The notion that positive and negative body image are distinct constructs is supported by findings that positive body image uniquely affects aspects of wellbeing and eating behaviors even after negative body image is accounted for (Avalos et al., 2005; Tylka and Wood-Barcalow, 2015a), and by evidence that individuals can simultaneously experience increases in both positive and negative body image (Tiggemann and McCourt, 2013). The realization that positive body image is a unique and complex construct that has protective effects on wellbeing has led scholars to advocate for its greater attention in the field (Avalos et al., 2005; Menzel and Levine, 2011; Tylka and Wood-Barcalow, 2015b; Grogan, 2016). We argue that positive body image should be integrated into the sociocultural model as a protective factor that prevents body dissatisfaction from leading to unhealthy behaviors, like disordered eating. First, we consider the construct of positive body image itself.

Positive body image is conceptualized as a holistic, multifaceted higher-order factor that is comprised of multiple lower-order dimensions (Menzel and Levine, 2011). However, the literature on the specific dimensions of positive body image is not well-developed and there is a lack of consensus on the number of facets that contribute to this construct (Halliwell, 2015). In their review of measures and methods for assessing positive body image, Webb et al. (2015) propose at least 10 constructs for positive body image: body appreciation, functionality, attunement (i.e., being responsive to body needs and engaging in mindful self-care), sanctification (i.e., perceiving the body as having spiritual significance and therefore deserving of respect), and pride, along with body image flexibility, a positive rational acceptance (i.e., coping with body-image related threats by engaging in rational self-talk and self-care), broad conceptualization of beauty (i.e., perceiving beauty in a diversity of body shapes and sizes as well as inner characteristics), self-perceived body acceptance by others, and positive body talk. However, the authors noted that many of these constructs have not been adequately conceptualized or operationalized explicitly within the context of positive body image, highlighting a gap in our understanding of how these facets may interact and influence the overarching construct. In the following review, we will focus on the nature and assessment of different aspects of positive body image: body appreciation, body functionality, and body compassion.

### Measuring positive body image

As research interest in and attention to positive body image has grown in the past two decades, so too has research on the best ways to identify individuals who display positive body image. There are several measures that tap into various facets of positive body image (Webb et al., 2015).

#### Body appreciation scale

By far the most comprehensive and widely used measure on positive body image is the Body Appreciation Scale (BAS; Avalos et al., 2005) and its updated counterpart (BAS-2; Tylka and Wood-Barcalow, 2015a). This unidimensional scale is intended to assess the extent to which one holds favorable opinions toward, accepts, and respects their body, as well as the extent to which they protect their body image by resisting the influence of media-promoted ideals (Avalos et al., 2005). The evidence-based revision from BAS to BAS-2 added updated items reflecting body appreciation, broad conceptualization of beauty, body acceptance, and inner positivity that influences self-care behaviors (Tylka and Wood-Barcalow, 2015a). The BAS-2 is inversely related to indices of negative body image, including body dissatisfaction, internalization of thin ideals, drive for muscularity, and body surveillance. Unlike measures of negative body image, the BAS-2 (like the original BAS), is purposefully ambiguous in its reference to the body, such that individuals are free to reference any aspect of the body (e.g., appearance, function, wellbeing, health) when responding to items (Tylka and Wood-Barcalow, 2015a). In further contrast to measures of negative body image, which typically focus on “lower-order” factors such as body shame or body dissatisfaction (Halliwell, 2015), the BAS-2 aims to assess the broader higher-order construct of overall positive orientation to the body. The BAS-2 was originally validated in college and community samples of women and men in the United States, demonstrating good construct validity, internal consistency, and test-retest reliability over a 3-week period (Tylka and Wood-Barcalow, 2015a). It has since been validated across a diverse range of cultures and has been translated to at least eight languages other than English, overall demonstrating strong psychometric properties and a consistent unidimensional structure, though some invariances have been noted across male and female genders (Argyrides, 2020; Razmus et al., 2020; Zarate et al., 2021). The scale has been adapted and validated for use in children as young as 9 years old (Halliwell et al., 2017) and a validated state-based version has been developed to capture momentary fluctuations in body appreciation across time and situational contexts (Homan, 2016).

#### Appreciation of body functionality

The construct of positive body image encompasses appreciation of one's whole body, not just in terms of its physical appearance but also in terms of its capabilities (Cash and Smolak, 2011; Tylka and Wood-Barcalow, 2015b). Researchers in the field of body image have predominantly focused on physical appearance and in doing so have neglected to adequately incorporate body functionality, with the latter describing everything the body can and is able to do (Cash and Pruzinsky, 2002). In their revision of the BAS-2, Tylka and Wood-Barcalow (2015a) included a newly developed item to specifically target functional appreciation (i.e., “I appreciate the pleasures and the functions my body provides for me, e.g., ability to walk, laugh, hug, etc.”) that was ultimately excluded from the final measure due to low factor loading. Although the BAS-2 is intended to be an encompassing measure of body image, this tool may not be adequately measuring one's appreciation of their body's functionality, given that participants might complete the survey with a view toward body aesthetics.

To address this gap, Alleva et al. (2017) developed the Functionality Appreciation Scale (FAS) with the aim of specifically assessing the extent to which one appreciates, honors, and respects the body for the functions it is capable of doing. The FAS consists of seven items, such as “I appreciate my body for what it is capable of doing” and “I am grateful for the health of my body, even if it isn't always as healthy as I would like it to be.” The scale has evidenced a unidimensional structure that is upheld across gender, and has been shown to have internal consistency, 3-week test-retest reliability, and criterion- and construct validity among samples of U.S. community men and women (Alleva et al., 2017). The scale has since been translated to at least three languages, and has been tested with several non-U.S. samples, overall demonstrating strong psychometric properties (Swami et al., 2019b, 2021; Cerea et al., 2021).

#### Body compassion

Altman et al. (2020) recently developed and validated the Body Compassion Scale (BCS) in two samples of undergraduate students. The scale was designed to measure compassion for the body in a way that amalgamated Cash's (2004) definition of body image mentioned at the start of this paper, and Neff's (2003b) conceptualization of self-compassion (Altman et al., 2020). The authors found the scale to have three factors or subscales: (a) defusion, or the ability to “unhook” from painful thoughts and emotions about the body, (b) common humanity, or the ability to recognize that one is not alone in experiencing negative thoughts and feelings about the body, and (c) acceptance, or the ability to accept the body while tolerating its flaws (Altman et al., 2020). Although the BCS has been relatively newly developed, it has recently been validated in other cultures, such as Italian women (Policardo et al., 2022) and Hong Kong adolescents (Wong et al., 2022). Although further evaluation of its psychometric properties is warranted, the BCS is a new tool that can be used to assess compassion for the body independent of general trait-level self-compassion. Even more recently, Beadle et al. (2021) developed and validated the Body Compassion Questionnaire (BCQ), which differs conceptually from the BCS as it purposely does not include the evaluative component of body image (i.e., one's evaluation of the body), and attempts to delineate the concept of body compassion from mindfulness. Derived from qualitative writing exercises, and incorporating Neff's (2003a,b) conceptualization of self-compassion and Gilbert's (2017) conceptualization of compassion, Beadle et al. (2021) determined their measure displayed a three-factor structure, with self-kindness, common humanity, and motivated action subscales, and that the BCQ was more predictive of disordered eating than general self-compassion. To our knowledge this scale has yet to be employed in other published research.

## Positive body image: Correlates, predictors, and outcomes

### Body appreciation correlates

The most widely studied positive body image construct is body appreciation, as measured by the Body Appreciation Scale (Avalos et al., 2005) and the revised Body Appreciation Scale-2 (Tylka and Wood-Barcalow, 2015a). Research indicates that body appreciation is the most central facet of positive body image (Swami et al., 2020), and is associated with numerous indices of psychological wellbeing such as subjective happiness, self-esteem, life satisfaction, optimism, proactive coping, self-compassion, and emotional intelligence (Avalos et al., 2005; Swami et al., 2010; Lemoine et al., 2018; Siegel et al., 2020). Behaviorally, body appreciation is also positively linked to a variety of pro-health indices including intuitive eating (i.e., eating according to internal hunger satiety cues), physical activity, women's sexual health and satisfaction, intentions to engage in skin cancer prevention activities, oral health status and behaviors, and willingness to receive COVID-19 vaccines (Dumitrescu et al., 2008; Tylka and Kroon Van Diest, 2013; Homan and Tylka, 2014; Gillen, 2015; Robbins and Reissing, 2018; Koller et al., 2020; Liu et al., 2021). Furthermore, body appreciation is negatively correlated with pathology, including disordered eating, maladaptive perfectionism, and neuroticism (Swami et al., 2008; Iannantuono and Tylka, 2012; Tylka and Wood-Barcalow, 2015b; Baceviciene and Jankauskiene, 2020). Studies of adult women suggest positive relationships between body acceptance by others, body appreciation, and intuitive eating (Avalos and Tylka, 2006; Augustus-Horvath and Tylka, 2011; Oh et al., 2012), and experimental research has shown that among women, trait-based body appreciation is protective against the harmful effects of exposure to sociocultural appearance ideals on body image, even among women who have high levels of internalized appearance ideals (Halliwell, 2013; Andrew et al., 2015).

A recent meta-analysis that examined gender differences in body appreciation found that men report higher levels of body appreciation than do women (He et al., 2020). However, the study also found that gender differences varied as a function of survey method, type of sample, and age of participants. Specifically, online studies tended to find greater gender differences on body appreciation than paper-and-pencil surveys. Further, and consistent with other research (Tylka and Wood-Barcalow, 2015a), gender differences on body appreciation were more pronounced among primary/middle/high school students as compared to adults. Finally, it was found that differences between men and women on level of positive body image decrease as adults age, as has also been found elsewhere (Tiggemann and McCourt, 2013).

### Functionality appreciation correlates

Although there are several important theories which incorporate the construct of body functionality (e.g., body conceptualization theory, objectification theory; Alleva and Tylka, 2021), for the purposes of this overview, we will focus on research that employed or discussed body functionality as measured by the functional appreciation scale (FAS; Alleva et al., 2017). As a more comprehensive review is outside the scope of the current paper, see Alleva and Tylka (2021) for an extensive review of theory, scales, and research related to functionality appreciation. Body functionality (as measured by the FAS) has been shown to be positively associated with engagement in sport (Soulliard et al., 2021), body appreciation, self-compassion (Alleva et al., 2017; Swami et al., 2019a), self-esteem (Swami et al., 2019, 2021, 2022; Linardon et al., 2020; Cerea et al., 2021; Namatame et al., 2022; Sahlan et al., 2022), life satisfaction (Namatame et al., 2022), proactive coping (Alleva et al., 2017), physical and mental health, gratitude (Swami et al., 2021; Namatame et al., 2022), body esteem (Cerea et al., 2021), body image flexibility (Linardon et al., 2020), and intuitive eating (Soulliard and Vander Wal, 2019; Namatame et al., 2022), and negatively associated with depressive symptoms (Linardon et al., 2020; Sahlan et al., 2022), body dissatisfaction, disordered eating/eating disorder symptoms (Soulliard and Vander Wal, 2019; Cerea et al., 2021; Swami et al., 2021, 2022), body surveillance (Cerea et al., 2021), drive for muscularity (Swami et al., 2021), drive for thinness (Cerea et al., 2021), overvaluation of shape and weight (Linardon et al., 2020), and general distress (Cerea et al., 2021).

Importantly, although functionality appreciation is not restricted to able-bodied individuals (Alleva and Tylka, 2021), an important area of future study will be further investigating the experiences of individuals with disabilities or body differences, as they may have a different relationship to body functionality (Vinoski Thomas et al., 2019; Rice et al., 2021). Indeed, qualitative research shows that women with visible physical disabilities have high levels of concern about the appearance of their bodies while performing body functions, including concerns about “looking disabled” to self and others (Vinoski Thomas et al., 2019). Additionally, more longitudinal studies examining the association between functionality appreciation and other positive body image constructs, disordered eating, and negative body image are needed.

### Emerging body compassion research

Body compassion (i.e., compassion extended toward one's body) is an aspect of positive body image which incorporates the constructs of body image and self-compassion. Although a relatively newly developed construct (see above description of the body compassion scale and body compassion questionnaire), there has been early promising cross-sectional research conducted on body compassion as measured by the body compassion scale (Altman et al., 2020). For instance, in a case study, Altman et al. (2017) described successfully targeting body compassion during the course of mindfulness and acceptance-based cognitive-behavioral therapy for body dissatisfaction. In a study among a sample of Portuguese community women, Oliveira et al. (2018) found that body compassion moderated the effect of general shame on body-related shame and disordered eating, lessening the impact of shame impact on both outcomes. In a similar sample of Portuguese community women, de Carvalho Barreto et al. (2020) concluded that self-compassionate actions (i.e., acting to relieve one's own suffering) and body compassion mediated the relation between a self-compassionate attitude and disordered eating attitudes/beliefs, such that general and body-specific compassion for the self were protective against problematic eating attitudes/behaviors. In a recent study of women with polycystic ovary syndrome, Van Niekerk et al. (2022) found that body image concern had a negative association with body compassion. Similarly, in a sample of Portuguese women, Barata-Santos et al. (2019) found that body compassion was negatively associated with binge eating symptoms. Although the development of ways to measure body compassion is promising, and early research supports its potential predictive utility, further research (particularly longitudinal) will need to be completed to determine its clinical and research utility over and above general self-compassion.

#### Research on positive body image

Overall, the evidence demonstrates robust associations between positive body image and multiple indices of psychological wellbeing, physical health, and self-care. However, because most of the studies on positive body image are correlational or cross-sectional, it is difficult to draw conclusions about causation and directionality. An overview of findings from prospective and experimental research studies is presented below, combining results regarding body appreciation and functionality appreciation, as studies often examine both.

##### Prospective studies

Prospective research has helped to identify both predictors and outcomes of positive body image and its facets (i.e., body appreciation). Specifically, a longitudinal cohort study by Andrew et al. (2016) found that among adolescent girls, body acceptance by others predicted an increase in body appreciation at 1 year, and that the reverse was also true, suggesting a bi-directional causal relation between the two constructs. Additionally, they found that body appreciation prospectively predicted increased levels of intuitive eating as well as decreased dieting behaviors, cigarette smoking, and alcohol consumption 1 year later. Taken together, the research suggests that promoting body appreciation in adolescent girls may increase the likelihood that they will adopt a healthy lifestyle as adult women.

Prospective research has also provided insight into the developmental trajectory of positive body image, with Gattario and Frisén (2019) tracking body image development from a negative body image in adolescence (age 10 years) to a positive body image in adulthood (age 24 years) among men and women. Results demonstrate that the trajectory of negative to positive body image varied greatly among individuals; however, most participants reported a generally positive body image by 18 years of age. From the qualitative data collected, three factors emerged as facilitating the development of positive body image: finding an accepting social context, the development of one's agency, and active use of strategies to improve body image. Further, the study identified gender differences regarding views of one's body. Specifically, women were more likely to consider positive body image as needing constant maintenance, while men were more likely to participate in appearance-enhancing activities aimed at improving body shape and were more likely to view themselves as resembling the male body ideal. These gender differences might be explained by the fact that puberty and body-related changes during adolescence move women away from feminine body ideals (i.e., women experience increases in overall body fat), whereas they move men toward body ideals (i.e., men experience increased muscularity).

##### Experimental studies

Experimental research has helped to identify ways in which positive body image may be cultivated. Guest et al. (2019) conducted a systematic review investigating positive body image interventions for adults and found that the intervention with the strongest evidence of effectiveness was a three-part writing exercise aimed at helping women focus more on their body functionality (Alleva et al., 2015, 2018a,b). Specifically, in a sample of young British women, Alleva et al. (2018a) applied the Expand Your Horizon intervention, a set of body functionality writing exercises, comparing it to an active control (creativity training). The authors found that women who completed the intervention displayed higher levels of appearance satisfaction, functionality satisfaction, and body appreciation, and these effects held through 1-month follow-up. In another evaluation of this short writing-based online intervention (i.e., Expand Your Horizon) among women with rheumatoid arthritis, Alleva et al. (2018b) found that compared to a waitlist control group, women who received the intervention showed increased functionality appreciation and decreased depression, with effects holding for the 1-month follow-up. Other outcomes such as pain-related disability and anxiety were not improved by the intervention. These results are in line with a previous successful application of this program, which also reported increases in appearance satisfaction, functionality satisfaction, and body appreciation in participants (Alleva et al., 2015). This intervention was shown to significantly increase body appreciation and body functionality satisfaction across these three separate studies, with gains maintained at 1 month (Alleva et al., 2018a,b). More recently, Alleva et al. (2021) asked undergraduates to write a letter to a friend appreciating either their friend's body functionality or their shared memories. Interestingly, both groups experienced an increase in state body functionality appreciation. These findings are promising, suggesting that relatively brief interventions aimed at helping women focus more on the many functions that their body performs can lead to improvements in positive body image.

In a review of body image interventions for children and adolescents (Guest et al., 2022), strong support was found for the use of a cognitive dissonance-based session with girls aged 14–15 years (Halliwell et al., 2015). Such programs teach participants how to verbally combat appearance and body ideals, and how to take personal action against them. As cognitive dissonance-based interventions are well-established in reducing negative body image, it is promising that such an intervention can simultaneously be used to promote positive body image in the form of body appreciation (Stice et al., 2008).

Several studies have examined body functionality as a potential protective factor against the consequences of viewing idealized media images. For example, Mulgrew et al. (2017) assigned women to either write positively about their body's appearance or functionality. Women in both conditions reported improved satisfaction with appearance and functionality, however, neither writing exercise was protective against the effects of exposure to idealized images of models. Mulgrew et al. (2019) conducted a similar follow-up study with two body functionality writing sessions before exposing women to idealized imagery 1-week later. Despite initial gains in functionality satisfaction, the exposure task resulted in reduced functionality satisfaction in both the intervention and control groups (Mulgrew et al., 2019). Similarly, Alleva et al. (2021) asked women to write about their body functionality or a control topic before they viewed idealized images. In contrast to Mulgrew et al. (2017, 2019) and Alleva et al. (2021) found that compared to the control group, women who wrote about body functionality had higher functionality satisfaction and body appreciation after viewing the images. Interestingly, other experimental research has demonstrated that state body appreciation can be increased in women by exposure to images of female models who do not conform to the thin-ideal body type and who have more “normal” looking bodies (Williamson and Karazsia, 2018). That same study, as well as others, also found that exposure to images of natural environments led to small but significant improvements in state body appreciation (Swami et al., 2018; Williamson and Karazsia, 2018).

## Self-compassion and its role in body image

Parallel to an increase in interest in positive body image as a concept that is important and distinct from body dissatisfaction has been a rise in thinking around ways to improve body image and not just treat body image disturbance. In this section, we discuss general, trait self-compassion and what is known about its role in body image. Compassion is defined as having an awareness of someone's suffering and being motivated to act to reduce that suffering (Goetz et al., 2010; Gilbert, 2019). There are two main theories of compassion for the self in the literature. The first theory was established from Buddhist teachings, and holds that self-compassion is comprised of three opposing states, self-kindness vs. self-judgment, common humanity vs. isolation, and mindfulness vs. overidentification (Neff, 2003a). Essentially, from this viewpoint, self-compassion consists of treating oneself as one would a good friend in times of suffering, with care and kindness (Neff and Germer, 2013). The second theory of self-compassion is rooted in an evolutionary approach, and states that compassion involves having an awareness of suffering and being motivated to act to reduce that suffering, whether that is directed toward others, or toward the self in the form of self-compassion (Goetz et al., 2010; Gilbert, 2019). From this viewpoint, compassion is postulated to have evolutionary roots, as it brings about cooperation in order to protect individuals in the community who are suffering or weak (Goetz et al., 2010). While shame is an emotional experience that marginalizes or separates people from others, compassion brings people together for support and connection (Gilbert, 2017). Thus, self-compassion is when a compassionate attitude, either one based in self-kindness (Neff, 2003a) or a commitment to alleviate suffering (Gilbert, 2019), is directed inward toward one's own suffering.

In the past 10 years, there has been significant progress in understanding how self-compassion buffers against negative attitudes and feelings about the body. Indeed, there is robust empirical evidence that self-compassion is protective against negative body image and disordered eating (e.g., Braun et al., 2016), is central to successful recovery from an eating disorder (e.g., Kelly et al., 2014a), and is negatively associated with shame (e.g., Ferreira et al., 2014). The following sections provide an overview of the state of the research on self-compassion and its relation to body dissatisfaction, disordered eating, and general/body shame. The research studies reviewed are organized by methodology.

### Cross-sectional studies

Self-compassion has been linked to lower levels of body image disturbance and eating pathology in non-clinical and clinical samples in a large number of cross-sectional studies. For example, in a recent correlational study with a mixed-gender non-clinical sample, Turk et al. (2021b) found that higher levels of self-compassion were associated with lower levels of eating pathology and body dissatisfaction through a pathway of comparative self-criticism (e.g., “I'm inferior to others”) followed by external shame. A cross-sectional study by Daye et al. (2014) found that retrospective recall of problematic caregiver eating messages was associated with higher levels of body shame and lower levels of self-compassion. Similarly, in an eating disorder sample, Ferreira et al. (2014) found that self-compassion moderated the relation between shameful memories and eating pathology, such that in individuals who had less intense shame memories, those with higher levels of self-compassion had lower eating pathology compared to those with low self-compassion. However, amongst individuals with more intense shame memories, levels of eating pathology remained high regardless of self-compassion level. Ferreira et al. (2013) found that among women with an eating disorder, higher self-compassion was associated with lower external shame and eating disorder symptoms, and higher levels of shame and body dissatisfaction predicted increased drive for thinness through reduced self-compassion. Conversely, in a community sample of women, the relation between external shame and drive for thinness was partially mediated by self-compassion, and the relation between body dissatisfaction and drive for thinness was not mediated by self-compassion. In a recent study of men, Maher et al. (2021) found that high levels of self-compassion predicted lower levels of body dissatisfaction, and that self-compassion moderated the relation between appearance ideal internalization and body dissatisfaction such that appearance ideal internalization and body dissatisfaction were more strongly associated when self-compassion was low. Cross-sectional research has also shown that individuals with clinical eating disorders such as anorexia nervosa and bulimia nervosa have lower levels of self-compassion and higher levels of fear of self-compassion compared to non-clinical samples (e.g., Kelly et al., 2014b). For example, in a sample of women with and without eating disorders, Kelly et al. (2014b) found that a high fear of self-compassion was most strongly related to eating pathology in the clinical group, whereas low self-compassion was most strongly related to eating pathology in the non-clinical group.

### Longitudinal studies

Although cross-sectional studies are the most common type of research design in the self-compassion literature, some recent longitudinal studies have been published examining the relation between self-compassion and body image/eating outcomes. For example, in a community sample of adult women, Turk et al. (2021a) found that higher levels of self-compassion were related to lower levels of eating pathology and body dissatisfaction 6 months later, and that external shame mediated this relation. In a 2-year study of undergraduate students, Stutts and Blomquist (2018) found that during the first year of college, self-compassion moderated the relation between weight and shape concerns and eating pathology, such that there was a stronger relationship between weight and shape concerns and disordered eating for participants who had low (vs. high) levels of self-compassion. However, these associations were not significant the following year. In a recent 3-year longitudinal cohort study of adolescent girls involved in sports, Pila et al. (2022) found that higher levels of both between- and within-person levels of self-compassion were associated with lower levels of body-related shame. Similarly, in a community adolescent sample, Pullmer et al. (2019) found that self-compassion was longitudinally associated with higher body satisfaction, and lower levels of disordered eating. Kelly and Tasca (2016) found that, in a clinical sample of patients completing inpatient or day hospital treatment for an eating disorder, following 3-week periods of increased shame, patients displayed higher levels of eating pathology. Periods of higher self-compassion were followed by lower levels of shame.

### Daily diary studies

Daily diary and ecological momentary assessment studies are a way to examine within-person self-compassion processes and their short-term associations with body image, shame, and eating disorder symptoms. In a daily diary study of a sample of individuals with anorexia nervosa, Kelly et al. (2020) found that on days when participants had more self-compassion compared to their typical baseline levels, they reported lower levels of eating pathology. Specifically, participants who had a mean self-compassion level that was average or higher had lower levels of eating pathology on particularly self-compassionate days, whereas in those with lower levels of self-compassion, self-compassion was not associated with eating pathology. Interestingly, there was no between-persons association between self-compassion and eating pathology. Similarly, in another daily diary study amongst undergraduate women, Kelly et al. (2016) found that on days where women were less self-compassionate than their mean levels, frequent contact with individuals who were body-focused was associated with higher levels of body image concerns, and lower levels of body appreciation and intuitive eating. This pattern held for both within and between persons analyses. Interestingly, on days when women were more self-compassionate than usual, more contact with body-focused others was associated with lower levels of body image concerns and higher levels of intuitive eating and body appreciation. In another daily diary study among female undergraduate students, Kelly and Stephen (2016) found that on days where women were more self-compassionate, they had more positive body image, engaged in less dietary restraint, and showed higher levels of intuitive eating. Between-person levels of self-compassion across the study days was associated with more positive eating and body image outcomes. In another daily diary study among female undergraduate students, Breines et al. (2014) found that participants reported lower levels of disordered eating behaviors on days they had higher levels of self-compassion toward their appearance. Additionally, responding to perceived body flaws with self-compassion was associated with less body shame and lower levels of disordered eating expectations. Among restrained eaters in a follow-up lab-based assessment, body shame mediated the relation between self-compassion and disordered eating (Breines et al., 2014).

### Qualitative studies

A number of qualitative studies have also explored the role of self-compassion in eating pathology and body image. For example, in a recent study of female and male undergraduates, Seekis et al. (2021) found that self-kindness (one component of Neff's conceptualization of self-compassion) was used by participants to cope with body-related distress in their daily lives, but only by those who already had relatively positive body image. Participants with poor body image were more likely to engage in self-judgment, with some expressing a fear of self-kindness. Although participants acknowledged the importance of common humanity (i.e., that one is not alone in their body image struggles), it was not used to cope with poor body image. Recently, a number of qualitative studies have explored the role of self-compassion in clinical eating disorder recovery. For instance, in a sample of 37 women with typical and atypical anorexia nervosa, Kelly et al. (2021) investigated participants' beliefs regarding the advantages and disadvantages of developing self-compassion as part of a self-compassion intervention. Perceived advantages included improved physical and mental health; general personal growth and improvement in coping skills; improved view of life and themselves, and healthier relationships. Perceived disadvantages included believing that self-compassion would lead to a drop in achievement; doubt regarding the genuineness and/or usefulness of self-compassion; and emotional challenges such as feeling undeserving of self-compassion.

In a series of studies that included individuals who were either currently in residential eating disorder treatment or were considered recovered from an eating disorder (Geller et al., 2021, 2022), current and former patients highlighted the importance of clinicians providing compassion to them, as well as clinicians openly modeling a self-compassionate stance toward themselves, as important in the process of treatment and recovery. In particular, participants noted how compassion from their therapist directly related to decreases in the experience of shame. Additionally, participants identified three processes that were integral to cultivating self-compassion during treatment: overcoming cognitive barriers to self-compassion (e.g., learning to see its utility), overcoming difficult life circumstances to prepare for implementation of self-compassion (e.g., recognizing and addressing unhelpful relationship patterns), and deciding to commit to the hard emotional work of treatment and make active efforts to change and learn to implement self-compassion (Geller et al., 2022).

### Systematic reviews

Finally, a few systematic reviews and meta-analyses have been published on the role of self-compassion in body image and eating pathology, pulling together the extant literature on the topic. In their systematic review, Braun et al. (2016) concluded that self-compassion was consistently associated with lower levels of eating pathology and body image disturbance in both clinical and non-clinical samples, although clinical samples tend to report lower levels of self-compassion than non-clinical samples. Recently, Turk and Waller (2020) conducted a meta-analysis to determine the relation between self-compassion and eating and body image outcomes, and found that self-compassion was negatively associated with eating pathology and body image concerns, and positively associated with positive body image, with medium to strong effect sizes.

In sum, the accumulated research on self-compassion provides compelling evidence that it is a fundamental concept for understanding the presence of eating disorder symptoms. Research across different labs and with different methodologies consistently shows that self-compassion is negatively associated with body dissatisfaction, positively associated with positive body image, and facilitates recovery from an eating disorder. Although there is less research on shame specifically, self-compassion has also been found to act as a buffer against shame's negative effects, particularly among women (e.g., Sick et al., 2020; Wang et al., 2020).

## Self-compassion interventions for body image and disordered eating

There has been a growing interest in self-compassion-focused interventions for eating and body image issues (e.g., Kelly and Carter, 2015; Kelly et al., 2017; Pinto-Gouveia et al., 2019; Kramer and Cuccolo, 2020). Proposed self-compassion interventions have been brief and one-off (e.g., Gobin et al., 2022) or longer-term. For instance, in one longer-term study, Kelly and Waring (2018) implemented a 2-week compassionate letter writing exercise in a sample of non-treatment seeking women with anorexia nervosa. Compared to their control condition, the letter-writing intervention produced greater increases in self-compassion and greater decreases in shame (external and body-focused) and fear of self-compassion. In a meta-analysis of randomized controlled trials of self-compassion-based interventions, Ferrari et al. (2019) concluded that self-compassion interventions lead to improvement across 11 domains, including eating behavior (large effect size), self-compassion, and self-criticism (moderate effect sizes). They did not specifically examine body image variables. In another meta-analysis, Turk and Waller (2020) concluded that self-compassion interventions for eating pathology and body image were effective compared to control groups.

In sum, self-compassion is a key construct that extends the sociocultural model of body image such that in the absence of self-compassion, the pathways between idealized body images, social comparison, internalization of body ideals, body dissatisfaction, eating disorders, and psychological distress can become well-established. Self-compassion appears to interrupt those pathways and protects people from body image disturbance. Body compassion may be an even more specific construct to focus on in order to prevent and treat body image distress.

## Conclusion and future directions

The sociocultural model of body image (i.e., The Tripartite Influence Model) remains a valid, evidence-based theoretical framework of body image. It is applied to explaining the social and individual pathways between societal body norms (e.g., idealized body images) to body dissatisfaction and associated behaviors and other forms of psychological distress. Societal norms around the types of bodies that are deemed to be attractive and desirable set off a chain of psychological reactions, resulting in body image disturbance and distress. The model has identified personality traits that make people especially vulnerable to negative body image resulting from comparison to idealized body norms, such an internalization of appearance ideals. The model has evolved somewhat with the literature from the past three decades. There are several issues still needing to be addressed, such as explaining the motives behind self-selected exposure to idealized body norms. It can be applied to men. However, we know of no research assessing the role of idealized body norms in the experiences of transgender or non-binary individuals. This is an important area for future research as it is not known whether the sociocultural model applies to non-cis-gendered individuals. Research on the effects of exposure to idealized body images has also largely excluded midlife and older adults. It is not known whether older adults compare themselves to idealized body norms characterized by youth. Because eating disorder prevention campaigns and body image treatments are informed by the sociocultural model of body dissatisfaction; it is imperative for research findings to build an accurate and nuanced framework for understanding the role of idealized body norms on body image in all genders across the lifespan. Three topics deserve more attention in this regard.

A related but largely parallel line of research to the sociocultural model has occurred around body shame. Whereas, body dissatisfaction can be conceptualized as an internal cognitive and emotional reaction to not meeting societal body ideals, body shame can be thought of as a deeper, internal and external emotional process that can also affect behaviors such as dieting and disordered eating. Future research should better delineate body dissatisfaction and body shame, as many clinicians and researchers may not be aware of the difference. We advocate for a heightened awareness of body shame among clinicians and suggest assessing for it in clients based on the validated measures described in the relevant section above. Furthermore, based on the emerging research, it seems likely that body shame is even more influential than body dissatisfaction in the development of clinical eating disorders such as bulimia nervosa. Whereas, body dissatisfaction is seen in both eating-disordered and non-eating-disordered populations alike, body shame is characterized by the emotional elements of disgust or anger at oneself that are especially harsh and self-critical.

At the same time as the sociocultural model has expanded and incorporated new findings, especially around personality risk factors and clinical eating disorders, the field has pivoted toward a more positive approach to the psychology of body image. Coinciding with the positive psychology movement, there is now ample evidence from the literature that positive body image is an important construct and is not simply the absence of body dissatisfaction. However, a focus only on the pathology of disordered eating will continue to ignore positive aspects of body image. Because body dissatisfaction is so common, especially among young women, we argue that attention to positive body image should be even more widely assessed and targeted clinically. It should be more widely recognized as a protective factor that can interrupt the pathways between body dissatisfaction and eating disorders. However, many researchers and clinicians pay little attention to positive body image. There is a very strong evidence base for clinicians to pay more attention to positive body image and fostering positive feelings toward one's body. Self-compassion may buffer people from body image disturbance by reducing social comparison and pressure to adhere to societal body ideals. More research is needed on whether self-compassion in general is better to foster as compared to body compassion more specifically.

Societal norms regarding idealized body types are ubiquitous in most societies. At present, social media may be the most influential communicator of body norms to people. By their nature, idealized body norms are exaggerated and unattainable for most people. It is well-documented that people who internalize the importance of having a “perfect” body and who compare themselves to idealized body norms have a more negative body image as a result. More research is needed to finetune the model in the digital age of social media and the motivation behind self-selected media exposure. More research is also needed to evaluate the utility of applying the sociocultural model to diverse populations (e.g., in terms of age, gender identity). The elimination of societal body ideals is unrealistic and current body ideals remain highly unattainable for the average person. More researchers and clinicians need to be aware of a particularly harmful component of body image to wellbeing: body shame. Measures of body shame should be considered for inclusion in future research on the effects of idealized body norms and body image to identify even more precisely the people who are most vulnerable to experiencing significant body image distress. In clinical contexts and for clients struggling with disordered eating, it is useful to assess for and monitor body shame. At the level of society, representations of truly diverse body sizes in media and advertising may help to lessen internalized beliefs around the correlation between having an idealized body type and being happy, attractive, or successful. At the level of the individual, self-compassion is the most well-researched method for mitigating the negative effects of idealized body norms and for fostering positive body image (e.g., body functionality appreciation) and wellbeing. Clinicians should become aware of and develop competence at delivering interventions to increase positive body image. To date, the most promising evidence-based method for increasing positive body image is through fostering self-compassion.

## Author contributions

All authors listed have made a substantial, direct, and intellectual contribution to the work and approved it for publication.

## Funding

This research was funded by a Social Sciences and Humanities Research Council of Canada (SSHRC) Insight Grant to JM.

## Conflict of interest

The authors declare that the research was conducted in the absence of any commercial or financial relationships that could be construed as a potential conflict of interest.

## Publisher's note

All claims expressed in this article are solely those of the authors and do not necessarily represent those of their affiliated organizations, or those of the publisher, the editors and the reviewers. Any product that may be evaluated in this article, or claim that may be made by its manufacturer, is not guaranteed or endorsed by the publisher.
